# A Review of Antiresonant Hollow-Core Fiber-Assisted Spectroscopy of Gases

**DOI:** 10.3390/s21165640

**Published:** 2021-08-21

**Authors:** Piotr Jaworski

**Affiliations:** Laser and Fiber Electronics Group, Faculty of Electronics, Wroclaw University of Science and Technology, Wybrzeze Wyspianskiego 27, 50-370 Wroclaw, Poland; piotr.jaworski@pwr.edu.pl

**Keywords:** antiresonant hollow core fibers, laser spectroscopy, wavelength modulation spectroscopy, tunable diode laser absorption spectroscopy, photothermal spectroscopy, photoacoustic spectroscopy, fiber gas sensors

## Abstract

Antiresonant Hollow-Core Fibers (ARHCFs), thanks to the excellent capability of guiding light in an air core with low loss over a very broad spectral range, have attracted significant attention of researchers worldwide who especially focus their work on laser-based spectroscopy of gaseous substances. It was shown that the ARHCFs can be used as low-volume, non-complex, and versatile gas absorption cells forming the sensing path length in the sensor, thus serving as a promising alternative to commonly used bulk optics-based configurations. The ARHCF-aided sensors proved to deliver high sensitivity and long-term stability, which justifies their suitability for this particular application. In this review, the recent progress in laser-based gas sensors aided with ARHCFs combined with various laser-based spectroscopy techniques is discussed and summarized.

## 1. Introduction

The end of the previous century has brought a new type of optical fiber, the so-called hollow-core fiber (HCF), which due to its unique structure and ability to guide light in the air via the photonic bandgap effect, rather than via the conventional total internal reflection phenomenon, revolutionized the development and application areas of optical fiber technology [1]. Further development of the HCF structure and exploration of different guidance mechanisms of light in the air have enabled access to the HCFs, which deliver a superb ability to efficiently guide laser light, especially in the mid-infrared (mid-IR) spectral band, where conventional solid-core fibers suffer from high attenuation of the glass material [2,3]. Currently, three major types of HCFs have been proposed, fabricated, and successfully used in various applications [4,5,6,7], amongst which the laser-based spectroscopy of gases has attracted significant attention of researchers around the world [8,9,10]: the hollow-core photonic bandgap fiber (HC-PBGF) [1], the Kagome HCF [11] and the Antiresonant Hollow-Core Fiber (ARHCF) [8]. Benefiting from an empty core, which can be filled with the target gas, HCFs can be utilized as low-volume absorption gas cells, forming versatile light-gas molecules interaction paths with the desired length within a sensor setup [8]. Since the sensitivity of the majority of laser-based gas sensors can be relatively simply and significantly enhanced by increasing the interaction path length, access to non-complex and long optical paths is highly desired. Hence, the incorporation of HCF-based absorption cells into laser-based gas detectors could lead to high sensor detection capability and less complex design in comparison with commonly used bulk optics-based solutions, e.g., utilizing multipass cells [12,13]. Multipass cells delivering optical paths with several tens of meters length require advanced optical arrangements for coupling into them the laser beam in a way allowing for obtaining the proper number of light passes, hence the desired path length. Since optical and optomechanical components are sensitive to vibrations and temperature changes, which negatively affect their long-term stability, even a slight misalignment of the coupling optics disturbs the light propagation inside the multipass cell. Unfortunately, optics-free coupling into a multipass cell is not possible. This leads to the increase in the noise level, the reduced amplitude of the measured signal, hence a significant drop in the detection capability of the sensor. On the other hand, light guidance in HCFs can be efficiently excited via an optics-free butt-coupling approach of the laser beam. Furthermore, the multipass cells based on the use of optical mirrors (e.g., Herriot- or White-type) mounted in e.g., metal optomechanical housings are sensitive to temperature changes due to thermal expansion of the material, which additionally affects the stability of the gas sensor. This can be minimized by using materials with lower thermal expansion coefficient, e.g., invar, however at cost of a significant increase in the sensor’s price, especially when multipass cells delivering several tens of meters long paths lengths are used in the setup.

HC-PBGFs have been successfully used in various gas sensor configurations, however, they target transitions of different gases in the wavelength range not exceeding 3.4 µm [9,14,15]. It was established that the main issues connected with the use of this particular type of fiber that significantly limits the sensitivity and versatility of fiber-based gas sensors arise from the multimode nature of these fibers and their maximum operational wavelength range [9,16]. Multimode guidance leads to the intermodal interference between the fiber-supported fundamental mode and the higher-order modes, which negatively impacts the noise level in the sensor [16]. This can be minimized by combining HC-PBGFs with spectroscopic techniques that have a built-in capability of reducing the impact of the fringe noise on the measured signal, e.g., Chirped Laser Dispersion Spectroscopy (CLaDS) or Photothermal Spectroscopy (PTS) [15,16]. Moreover, despite guidance in air, this fiber is still characterized by a relatively high overlap between its glass structure and the guiding light, which limits the transmission bandwidth to approximately 3 µm spectral band [17]. Furthermore, due to the small core size (typically up to 20 µm), the gas filling time of HC-PBGFs can reach even several hours, which severely limits the response time of the sensing systems utilizing these fibers [16].

A partial solution to the issues that are present in HC-PBGFs comes with the aid of the Kagome type HCFs, which guide light via the inhibited coupling mechanism [11]. As a result of a modified fiber structure and different light guidance principles, these fibers can efficiently transmit light in the near-infrared (near-IR) and mid-IR [11,18]. Furthermore, the core size of the Kagome fibers is a few times greater (116 µm) in comparison with the HC-PBGFs guiding light within the same spectral band, which results in the reduction of the gas filling time down to several seconds [18]. Nevertheless, it was indicated in [10,18] that the problem connected with the multimode guidance is also present in the Kagome HCFs, which was identified as the main limiting factor in the performance of the gas sensors utilizing these fibers.

**Figure 1:**
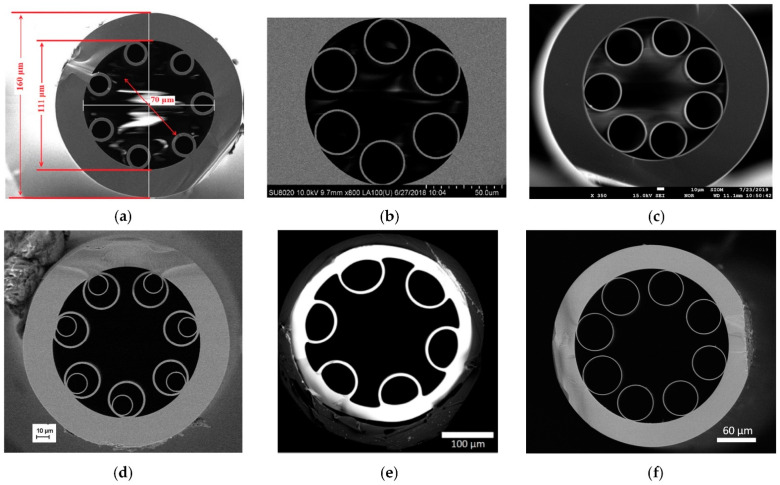
Examples of ARHCFs used in gas sensing systems. (**a**) Silica-based ARHCF is designed to operate in the 2 µm wavelength range with a core size of 70 µm. Reprinted with permission from [21] © The Optical Society. (**b**) Six-capillary cladding silica-based ARHCF with a core diameter of 65 µm for guidance at ~1.55 µm and 3.34 µm. Reprinted with permission from [26] © The Optical Society. (**c**) Seven-capillary cladding silica-based ARHCF with a core size of 84 µm providing low-loss transmission in the near- and mid-IR. Reprinted with permission by MDPI from [8]. (**d**) Silica-based ARHCF with nested capillary cladding and a core diameter of 65 µm for guidance at ~4.54 µm. Reprinted with permission from [27] © The Optical Society. (**e**) Tellurite ARHCF enabling light guidance ~5 µm inside a hollow core with a 139 µm diameter. Reprinted with permission from [28] © The Optical Society. (**f**) 5.26 µm-guiding borosilicate glass-based ARHCF with a core size of 122 µm.

ARHCFs, in which light transmission is realized by the Antiresonant Reflecting Optical Waveguiding (ARROW) principle [19], can deliver low loss in both near- and mid-IR spectral bands, fast gas exchange time, and single-transversal mode operation if a proper fiber structure is designed [8,20]. Currently, ARHCFs have been successfully used in gas sensors utilizing a variety of laser-based sensing techniques, i.e., Tunable Diode Laser Absorption Spectroscopy (TDLAS), Wavelength Modulation Spectroscopy (WMS), PTS, and Photoacoustic Spectroscopy (PAS) [8,21,22,23]. Examples of the ARHCFs used in gas sensing applications are depicted in Figure 1. Researchers have shown that the ARHCF-aided gas sensors can target molecules with transitions in the wavelength range up to 5.26 µm, which is unreachable with the use of other types of HCFs [20,24]. Furthermore, benefiting from their ability to simultaneously guide laser radiation within two dissimilar spectral bands, the ARHCF-based detectors can be used to analyze gas mixtures that contain molecules having transitions in both near- and mid-IR [8]. Similar to the Kagome HCFs, ARROW-guiding fibers are characterized by the core size in the range of several tens of micrometers, which in combination with a proper gas delivery system allows obtaining gas exchange times in the range of several seconds [20]. The combination of ARHCF-based gas absorption cells with, e.g., the PTS technique enables obtaining superb long-term stability of the sensor, giving a promising perspective for their future application in out-of-lab conditions [25]. The sensors utilizing such fibers have been demonstrated to provide detection capability even at a level comparable to the bulk optics-based setups, indicating that the fiber-based configuration of the sensors can form a new branch of sensitive, selective, and non-complex gas sensing platforms.

In this review, the recent progress in ARHCF-based gas sensors utilizing the aforementioned gas sensing techniques will be discussed. Several different sensor configurations are presented and their advantages along with main limiting factors are reviewed. Section 2 of this review aims at explaining the light guidance properties of the ARHCFs. Section 3 is devoted to the implementation of the ARHCFs into TDLAS-based gas sensors. Section 4 is focused on the WMS gas sensors aided with different types of ARHCFs. Section 5 presents the PTS technique supported by the ARHCFs and explains how the few-moded guidance of the fiber can be transferred to the high sensor stability and sensitivity together with an introduction to the new gas sensing method in ARHCFs, the so-called Photoacoustic Brillouin Spectroscopy (PABS) [23]. Section 6 summarizes the performance of the reported up-to-date ARHCF-aided gas sensor configurations.

## 2. Light Guidance in Antiresonant Hollow-Core Fibers

**Figure 2:**
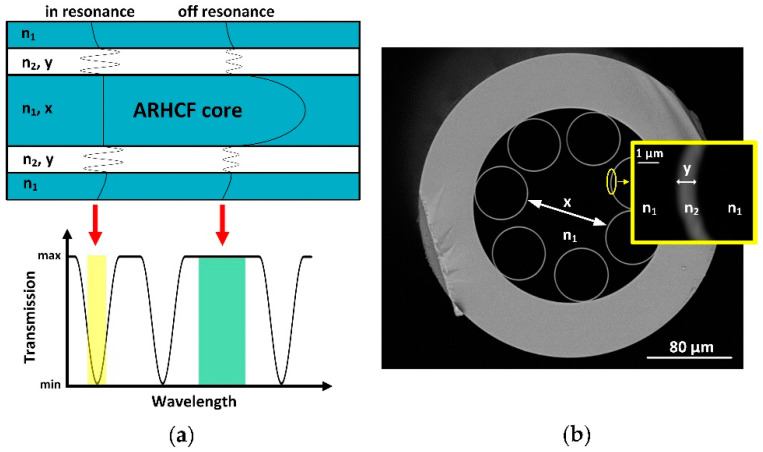
Light guidance mechanism in ARHCF. (**a**) 2D representation of the ARHCF (top) showing light transmission (bottom) in the core while the coupled light wavelength is in resonance and off resonance with the core wall. When the optical frequency (wavelength) does not match the resonant frequency of the Fabry–Perot cavity, the transmission of light in the core reaches its maximum. (**b**) SEM image of the ARHCF designed to operate at ~3.4 µm wavelength with a core wall thickness of ~1 µm. The inset shows the core boundary layer forming the resonant cavity. n_1_ and n_2_—refractive indices of the air region (core, gaps between capillaries and inner parts of the capillaries) and capillary walls, respectively, x—core diameter, y—capillary wall thickness.

ARHCFs are a new type of HCFs, which light guidance mechanism can be explained by the means of the ARROW model as shown in Figure 2a. According to this, the core boundary area of the ARHCF can be treated as a Fabry–Perot resonant cavity, as it is formed by low and high refractive index layers (e.g., air and glass) as presented in Figure 2b [8]. This Fabry–Perot cavity enables only the transmission of the optical frequencies, which are not in resonance with the core wall (capillary walls). These optical frequencies are reflected back to the fiber core where they propagate with low loss. On the other hand, the resonant optical frequencies cannot be confined within the fiber core and leak away to the cladding area where they experience high leakage and material loss [8]. The antiresonant wavelength range supported by an ARHCF can be calculated according to the following formula [19]:

(1) $$\lambda_{antires}=\frac{4y}{\left(2m+1\right)}\sqrt{n_{2}^{2}-n_{1}^{2}},\;m=0,1,2,\;\ldots$$

where *y* is the core wall thickness (capillary wall thickness), *n*1 and *n*2 are the refractive indices of the core and cladding, respectively. The resonant wavelength range can be defined as [19]:

(2) $$\lambda_{res}\sim \frac{2y}{m}\sqrt{n_{2}^{2}-n_{1}^{2}},\;m=0,1,2,\;\ldots$$

Based on the above equations, it can be concluded that the transmitted wavelength, and thus the position of the transmission window, depends mainly on the thickness of the capillary walls and not on the core size. However, it was reported in [29] that the hollow-core diameter of the ARHCF and diameter of the cladding capillaries have a strong impact on the bending properties and single-mode guidance of this particular fiber type. It was shown that dimensions of both have to be carefully selected to match the optimum ratio of core/capillary diameter of ~0.65 that enables single-mode transmission within the fiber low-loss window as a result of increased loss ratio between the fundamental mode and the higher-order modes supported by the fiber [8,29].

ARHCFs are fabricated with the aid of the commonly used stack-and-draw technique [30]. In majority, these fibers are drawn down from high purity fused silica glass (e.g., Suprasil F300) [8], however, due to the high material absorption of this material at the wavelengths above 5 µm, several successful attempts have been reported on fabricating ARHCFs from borosilicate and telluride glass allowing these fibers to efficiently guide light beyond the aforementioned wavelength range [28,31]. Thanks to the unique structure and light guidance properties, ARHCFs deliver better performance and versatility in comparison to other types of HCFs, especially in the area of fiber-aided gas sensing. A comparison of the parameters of the most commonly used HCFs in gas sensing is presented in Table 1.

## 3. Tunable Diode Laser Absorption Spectroscopy

One of the simplest and easiest methods used for laser-based gas sensing is the TDLAS [32,33]. In TDLAS, the information about the molecular concentration within a defined measurement path is retrieved based on the analysis of an interaction between the laser radiation and the gas molecules. The interaction leads to the absorption of light by the gas molecules, which are excited at the wavelength corresponding to the selected molecular transition. This phenomenon is governed by the Beer–Lambert law and expressed by the following formula [34,35]:

(3) $$\frac{I_{p}}{I_{0}}=\exp\left(-\varepsilon \left(\lambda \right)L\right),$$

where I_p_ corresponds to the light intensity after passing through the gas sample, I_0_ is the incident light intensity, ε represents the absorption coefficient of the gas molecules, λ is the wavelength of the light expressed in wavenumbers and L is the light-gas molecules interaction path length. In TDLAS-based gas sensors, the molecules of the target gas are typically illuminated by light delivered from a narrow linewidth laser, e.g., a distributed feedback diode laser (DFB) or a quantum cascade laser (QCL). The level of absorption of the gas molecules excitation light is observed as a drop in the signal intensity registered by a photodiode, while the laser beam is passing through the gas sample and its wavelength is tuned across the gas transition or kept at its peak. According to Equation 1, the sensitivity of the sensors relying on this method can be easily and efficiently enhanced by increasing the interaction path length within the sensor’s setup. This is commonly realized by implementing bulk optics-based absorption cells or multipass cells, e.g., Herriot-, White- or toroidal-type, which are filled with the measured gas sample [12,36,37]. This approach indeed results in the improved sensor’s performance, however, at the cost of the significantly increased complexity of its configuration and reduced immunity to, e.g., vibrations, temperature drifts, etc. Therefore, the application of the HCFs, especially the ARHCFs, seems to be a promising way to deliver low-volume, robust, and long optical paths. A successful demonstration of TDLAS-based gas sensors aided with ARHCFs has been already demonstrated by several research groups justifying the viability of this approach [21,24,35,38].

**Figure 3:**
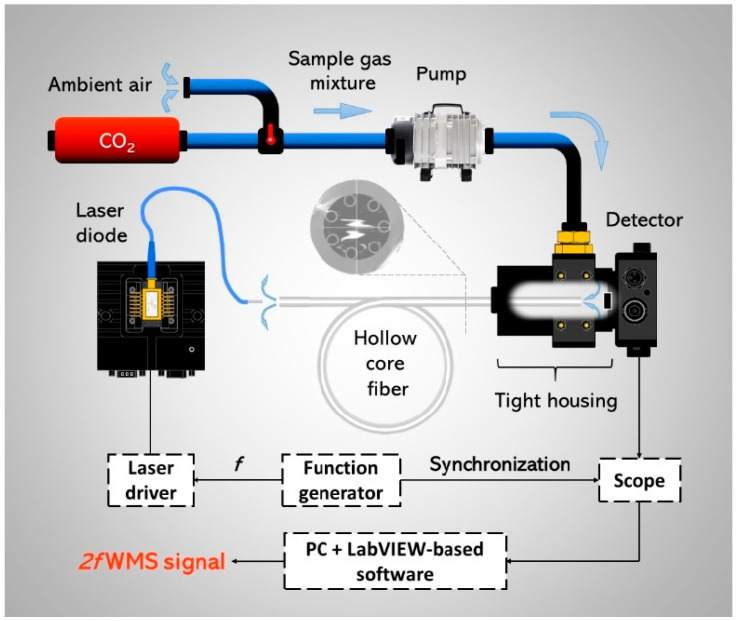
A schematic representation of the TDLAS/WMS gas sensor utilizing a 1.35 m long absorption cell based on an ARHCF. Reprinted with permission from [21] © The Optical Society.

Nikodem et al. reported in [21] a very simple carbon dioxide (CO_2_) sensor configuration utilizing a silica-based 7 capillary ARHCF as depicted in Figure 3. CO_2_ molecules were excited using a fiber-coupled discrete mode diode laser targeting their strong absorption line at 2.004 µm. The absorption cell within the sensor setup was formed by a 1.35 m long ARHCF with a hollow core diameter of 70 µm as depicted in Figure 1a. The fiber output end facet was placed in an air-tight housing used as a gas-filling cell, which was closed with a photodetector. The light from the laser was directly coupled into the ARHCF using a simple butt-coupling method. Subsequently, the fiber-delivered beam was directed onto the photodetector using a similar approach. Hence, the sensing part of the sensor was constructed in an all-fiber configuration. The ARHCF was filled with the target gas using a slight overpressure of 100 Torr. Despite a significantly reduced sensor complexity, the proposed system was characterized by a very poor detection capability. It allowed registering clear spectroscopic signals arising from only high (at the level of 1.5%) concentrations of CO_2_ inside the ARHCF. This was a direct result of the high background noise level induced by the optical fringes arising from the light coupling method used, intermodal interference in the ARHCF, and its non-uniform guidance characteristic. On the other hand, the responsivity of the sensor was at the level of several seconds, which is more than two orders of magnitude faster in comparison to the detectors based on the use of conventional HC-PBGFs [39].

**Figure 4:**
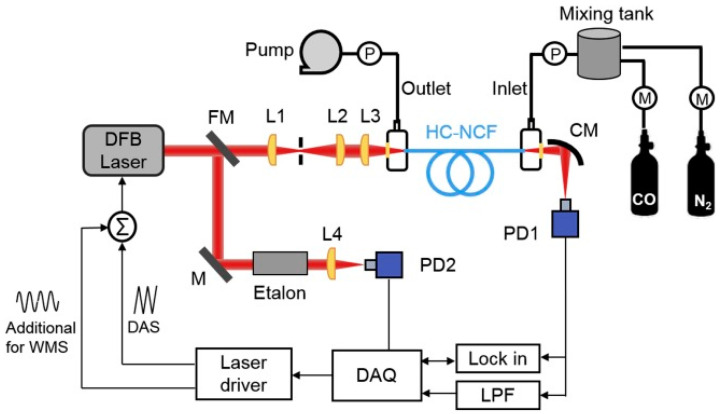
A setup of the CO sensor relying of the use of the TDLAS technique and an ARHCF-based gas absorption cell. L—lenses, M—mirror, CM—concave mirror, FM—flip mirror, P—pressure gauge, LPF—low-pass filter, PD1/PD2—photodetectors, DAQ—data acquisition card. Reprinted from [38] with permission from Elsevier.

Another interesting TDLAS-based gas sensor configuration shown in Figure 4 was reported by Yao et al. in [38]. The gas absorption cell in the setup was formed by a 0.85 m long ARHCF with an air-core diameter of 40 µm, which both ends were placed in air-tight gas filling cells closed with calcium fluoride (CaF_2_) windows. The ARHCF was filled with carbon monoxide (CO) using an overpressure of 0.8 bar, which resulted in the gas exchange time of the sensor at the level of 5 s. CO molecules inside the ARHCF core were excited with the aid of a 2.3 µm DFB laser, which was coupled into the fiber core using a set of properly selected lenses with a coupling efficiency of 90%. The minimum detection limit (MDL) in this particular sensor configuration reached 13 parts-per-million by volume (ppmv) of CO, yielding the noise equivalent absorption (NEA) of 5.2 × 10^−6^ cm^−1^. Similar to the earlier described work, the main limiting factor of the sensor was related to the presence of modal noise in the fiber, indicating not sufficient suppression of the higher-order modes along the relatively short fiber length and not entirely optimized light coupling conditions into the ARHCF core.

Further work in this area reported by Yao et al. in [35] was focused on developing a TDLAS-based sensor targeting a strong transition of nitrous oxide (N_2_O) in the mid-IR spectral band. The sensor configuration was similar to the one presented in Figure 4, however, with the main difference in the type of the ARHCF and the used laser. In this case, the N_2_O molecules were excited at 2778.37 cm^−1^ (~3.6 µm) using an interband cascade laser (ICL), which output was coupled into the gas-filled ARHCF with a coupling efficiency of 66%. The gas molecules-light interaction path was formed by a six-capillary cladding ARHCF with a core size of 65 µm and a length of 120 cm, which was filled with N_2_O via a pair of gas cells placed at its ends aided with an overpressure. Despite low transmission loss at the level of 0.6 dB/m at the considered wavelength, the fiber was characterized by a few-moded behavior that influenced the overall performance of the sensor. It was noticed that both the fundamental mode and the higher-order modes were simultaneously excited in the fiber, leading to the intermodal interference, which directly impacted the noise level in the registered spectroscopic signal, hence reducing the sensors’ detection capability. Nevertheless, the authors have shown that this parasitic effect can be minimized by properly selecting the light coupling conditions into the ARHCF and reducing the pressure of the gas inside the fiber core. As a consequence, the sensor reached an NEA of 2.5 × 10^−7^ cm^−1^.

The most recent work published by Yao et al. in [24] was focused on developing a TDLAS-based gas sensor utilizing a tellurite glass-based ARHCF enabling efficient light guidance above 5 µm wavelength. The 21 cm long ARHCF consisted of six non-adjacent capillaries forming its cladding and defining the hollow core region with a diameter of 75 µm, similar to the fiber shown in Figure 1e. The input end facet of the ARHCF forming the absorption cell was placed inside a pressure-tight gas cell closed with a CaF_2_ wedged window, which was implemented to reduce parasitic interference. The gas cell was mechanically modified in a way that allowed it to be easily connected to the cage system rods, hence properly and efficiently align the fiber end placed in the cell with respect to the focusing lens and a QCL. This approach provided a robust and stable coupling between the laser and the fiber. Similar to the previously described work, the ARHCF was filled with the target gas using an overpressure, which allowed obtaining the response time of the sensor below 1 s. The nitric oxide (NO) molecules inside the hollow core of the fiber were pumped at 5.26 µm using a continuous wave (CW) light from a QCL. The registered TDLAS signal from 100 ppmv NO in the ARHCF was characterized by the presence of strong background noise, which resulted from the multimode nature of the fiber, and could not be eliminated by simple signal averaging or the usage of different light coupling conditions. However, the authors minimized its influence on the sensor’s performance by introducing proper electrical filtering of the signal at the frequencies where the fringe noise was dominating. Thanks to this, the sensor reached an MDL of 1.2 ppmv, which corresponds to an NEA of 2.1 × 10^−5^ cm^−1^.

## 4. Wavelength Modulation Spectroscopy

WMS technique is a modification of the conventional TDLAS method, which enables reducing the influence of noise on the measured spectroscopic signal. In WMS, the wavelength of the laser that is used to excite gas molecules is (in comparison to TDLAS) additionally modulated with a sinusoidal signal with a strictly defined frequency and modulation depth, both dependent on the target gas transition characteristic [40,41]. The modulated laser frequency and the spectral absorbance in WMS are described by the following equations [41]:

υ(t) = ῡ + Acos(2πft),(4)

(5) $$-\alpha \left[ῡ+\mathrm{Acos}\left(2\pi \mathrm{ft}\right)\right]=\sum_{k=0}^{\infty }H_{k}\left(ῡ,A\right)\cos\left(k2\pi \mathrm{ft}\right),$$

(6) $$H_{k}\left(ῡ,A\right)=\frac{P_{X_{i}}L}{\pi }\int_{-\pi }^{\pi }\sum_{j}S_{{j\varphi }_{j}}\left(ῡ+\mathrm{Acos}\theta \right)\cos k\theta d\theta ,$$

where υ(t) is the modulated laser frequency in function of time, ῡ is the center laser frequency, A corresponds to the modulation depth, f is the modulation frequency, α is the spectral absorbance, P is the total gas pressure, X_i_ is the mole fraction of the absorbing gas sample, S_j_ is the j-th absorption line strength function and φ_j_ is the j-th absorption line shape function and L defines the interaction path length. Typically, the modulation frequency is in the range of a few to a few tens of kHz with a modulation depth equal to ~2.2 × full width at half maximum (FWHM) of the selected gas absorption line. In WMS-based sensors, the sinusoidally modulated laser beam experiences a nonlinear interaction with the gas molecules. This leads to the rise of additional components in the signals registered by the photodetector at frequencies corresponding to the harmonics of the fundamental modulation frequency. The amplitude of the even harmonics is proportional to the concentration of gas molecules within the measurement path length, hence the sensitivity of such sensors can be effectively increased by elongating the gas-laser interaction path. The harmonic components can be efficiently retrieved using a phase-sensitive lock-in amplifier-based approach [20,40]. As the lock-in amplifier allows demodulation of the measured signal at the desired frequency with a limited demodulation bandwidth, the noise level, which manifests itself especially in the lower frequency range, can be reduced. Therefore, the signal-to-noise ratio (SNR) of the sensor can be significantly increased in comparison to the TDLAS-based technique, which directly enhances the detection capability of the gas spectrometers [24].

It has already been demonstrated by various research groups that a combination of the WMS technique with ARHCFs leads to a significant improvement in the sensor’s detection capability, which results from the reduction of the fringe noise [21,24,38]. When the configurations of the sensors described in Section 3 of this manuscript were modified to allow WMS-based signal acquisition, the obtained NEA values were decreased by even two orders of magnitude compared to sensors operating in the pure TDLAS regime [24]. This enabled the ARHCF-based gas sensors to reach detection limits at a level comparable to the state-of-the-art bulk-optics-based setups.

**Figure 5:**
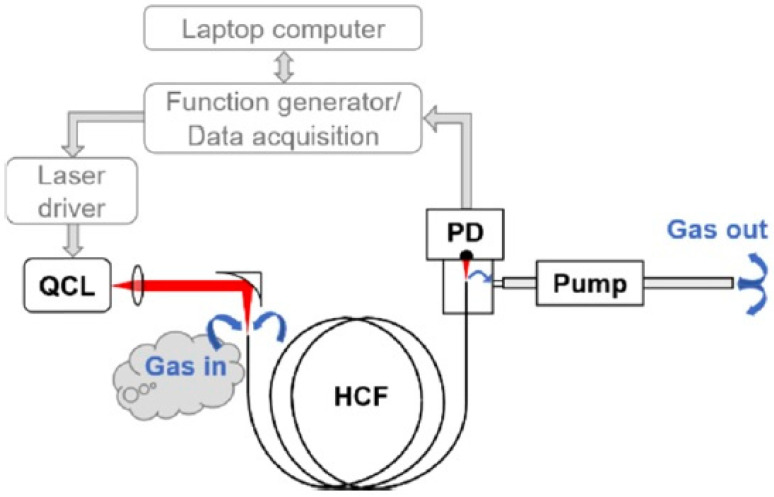
Experimental setup of the ARHCF-based N_2_O sensor utilizing WMS technique and a 4.54 µm QCL. Reprinted with permission from [27]. © The Optical Society.

Especially interesting work focused on WMS-based gas sensing aided with ARHCFs concerns the recent development of these fibers, which enabled them to guide light above 4.5 µm wavelength range. Nikodem et al. reported in [27] the first experimental demonstration of an ARHCF-based system capable of targeting a very strong N_2_O absorption line located at 2203.7 cm^−1^. As presented in Figure 5, the sensor utilized a QCL as a gas excitation source, which wavelength was tuned to the center of the selected N_2_O transition and subsequently coupled via an off-axis parabolic mirror into an absorption cell formed by a 3.2 m long nested ARHCF (shown in Figure 1d). The gas delivery system and method were similar to the ones described earlier and allowed filling the fiber core within the 23 s period. The sensor in this configuration reached an MDL of 5.4 ppbv at 1 s integration time, which corresponds to a minimum fractional absorption (MFA) of 1.2 × 10^−4^ (NEA ~3.7 × 10^−7^ cm^−1^). The obtained detector’s sensitivity was not at the record level, mainly due to the transmission characteristic of the fiber at the considered wavelength range. In this configuration, the QCL wavelength was placed at the edge of the low-loss transmission band of the fiber, where ARHCFs are typically characterized by the few-moded behavior [4]. Nevertheless, due to its unique structure, the fiber was characterized by an exceptional immunity to bending, which indicates the excellent robustness, compactness, and versatility of the ARHCF-based absorption cells delivering a few meters long interaction path.

**Figure 6:**
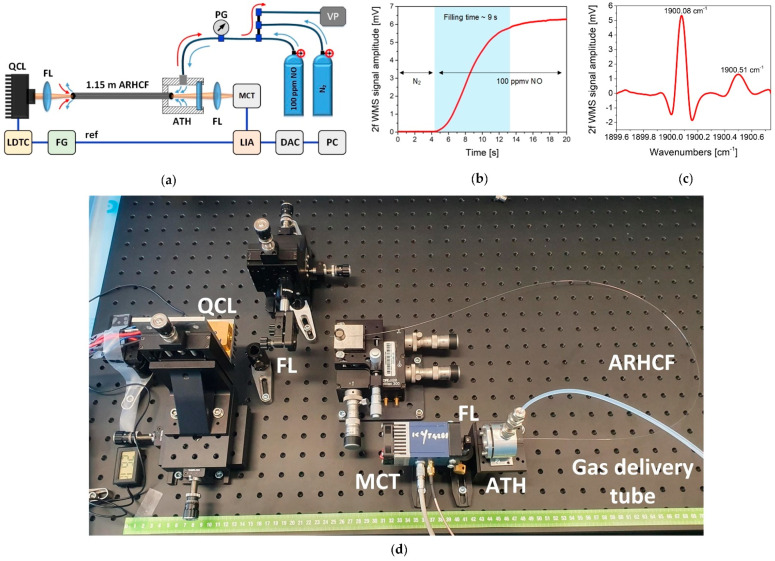
NO sensor operating at 5.26 µm based on the use of the WMS technique and a borosilicate ARHCF. (**a**) Experimental setup. QCL—quantum cascade laser, LDTC—laser driver, FL—focusing lens, FG—function generator, ATH— air tight housing, MCT—mercury-cadmium-telluride photodetector, PG—pressure gauge, VP—vacuum pump, LIA—lock-in amplifier, DAC—data acquisition card, PC—computer. (**b**) Gas filling profile of the ARHCF using an overpressure-assisted gas delivery method. (**c**) 2f WMS signal spectrum of the NO doublet for 100 ppmv NO inside 1.15 m ARHCF. Reprinted with permission from [20] © The Optical Society. (**d**) Photograph of the sensor setup.

The operational wavelength range of the ARHCF-based gas sensors was significantly increased with the development of borosilicate-glass- and telluride-glass-based fibers [28,31], which broke the barrier of 5 µm wavelength range, where the attenuation of silica glass increases rapidly [3,42]. Thanks to this unique feature, the ARHCF technology could be implemented in NO detectors. Jaworski et al. reported in [20] the first experimental demonstration of a WMS-based NO sensor utilizing a 1.15 m long borosilicate glass ARHCF, as depicted in Figure 6a. The sensor targeted a strong NO doublet located in the vicinity of 1900.08 cm^−1^, which was registered with the aid of a QCL. The gas filling method was similar to the one described earlier. Thanks to the large core size of the ARHCF (122 µm diameter), the sensor was characterized by the filling time of less than 10 s as shown in Figure 6b. As a result of the low-loss and single-transversal-mode guidance of the fiber, the sensor reached an MDL of 20 parts-per-billion by volume (ppbv) for 70 s integration time, which yields an NEA of 2.0 × 10^−5^ cm^−1^ and allowed registering clear spectra of 2f WMS signals from 100 ppmv NO inside the fiber as plotted in Figure 6c. Figure 6d shows a photograph of the sensor, which length does not exceed 75 cm. It is expected that the size of the sensor could be further reduced by decreasing the size of the electronic and optomechanical components used together with tightened bending of the fiber-forming the absorption cell. The authors indicated that the obtained MDL was less than an order of magnitude worse in comparison to a sensor utilizing the more advanced and complex quartz enhanced PAS technique [43]. This result was further improved by Yao et al. as reported in [24], where the WMS-operating sensor utilizing a tellurite ARHCF reached an MDL of 6 ppbv for 30 s integration time.

**Figure 7:**
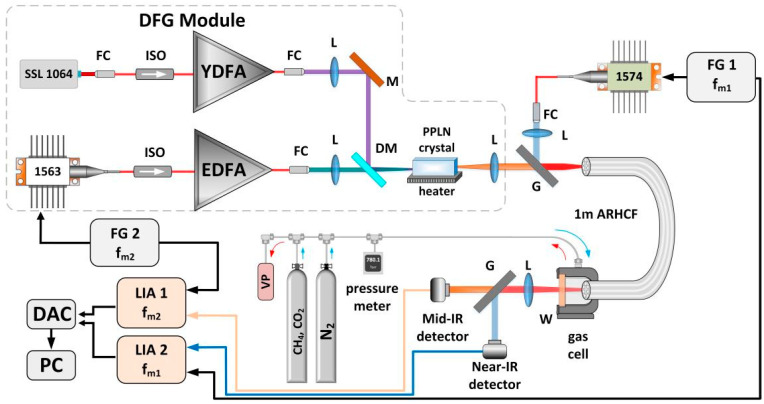
Schematic of the dual-gas sensor based on the near- and mid-IR guiding ARHCF and WMS technique. SSL—diode pumped solid state laser, FC—fiber collimator, ISO—isolator, YDFA/EDFA—Ytterbium- and Erbium-doped fiber amplifiers, L—lenses, M—mirror, DM—dichroic mirror, G—germanium window, W—CaF_2_ wedge, FG—function generator, LIA—lock-in amplifier, DAC—data acquisition card, PC—computer, VP—vacuum pump. Reprinted from [8] with permission from MDPI.

Another interesting and highly advantageous feature of the ARHCFs concerns their unique ability to transmit with low-loss light in several dissimilar wavelength bands. Jaworski et al. utilized this phenomenon for gas sensing and for the first time demonstrated the simultaneous detection of CO_2_ and methane (CH_4_) inside the ARHCF, targeting the transitions of these gases in the near- and mid-IR spectral bands [8]. The experimental setup shown in Figure 7 consisted of a difference frequency generation (DFG) and DFB sources, which operated at 3.334 µm and 1.574 µm, respectively. The DFG source was used to excite molecules of CH_4_, while the DFB laser targeted the CO_2_ transition. Both lasers were simultaneously coupled into a 1 m long ARHCF (shown in Figure 1c) filled with a mixture of the aforementioned gases through a gas filling cell. Thanks to the low loss and near single-mode guidance of the fiber at both wavelengths, the sensor reached an MDL of 24 ppbv for 40 s integration time and 144 ppmv for 1.5 s integration time for CH_4_ and CO_2_, respectively. The obtained MDLs yielded NEA coefficients of 1.6 × 10^−7^ cm^−1^ (CH_4_) and 1.17 × 10^−7^ cm^−1^ (CO_2_). The sensitivity of the sensor beats the performance of the WMS-based setups utilizing Kagome HCFs and HC-PBGFs, confirming the versatility of this approach [14,18]. The authors indicated that the further improvement of the developed sensor’s sensitivity could be obtained by introducing a longer fiber (i.e., with several tens of meters in length) with a properly modified structure, allowing obtaining pure single-mode transmission and uniform loss within the guidance windows.

## 5. Photothermal and Photoacoustic Spectroscopy

PTS is a technique where the spectroscopic signal retrieval is directly related to the heating of gas molecules with the aid of laser light [44,45]. In PTS, the gas molecules are excited with a laser source (*pump*) that emits radiation at a wavelength matching the selected gas transition (similarly to TDLAS and WMS). However, the retrieved signal is not connected with the intensity drop of the laser due to its gas-induced absorption, but with the local refractive index (RI) change that results from the increased temperature of the gas sample due to non-radiative relaxation of the molecules illuminated by the *pump* light [44]. The observed change of the RI can be determined based on the following equation [46]:

(7) $$\Delta n=\frac{\left(n-1\right){\varepsilon P}_{\mathrm{exc}}}{T_{0}4{\pi a}^{2}{\rho C}_{p}f},$$

where n and ε are the refractive index and absorption coefficient of the gas sample, respectively, P_exc_ is the intensity of the *pump* light, T_0_ is the absolute temperature, a is the *pump* beam diameter, ρ is the gas sample density, C_p_ corresponds to the specific heat of the gas sample, f is the modulation frequency of the *pump* light. The photothermal-induced RI change is typically retrieved using the second laser—*probe* (with a wavelength different from the *pump* light), usually using an interferometric approach. The *probe* light due to RI change experiences a phase shift according to the following formula [44]:

(8) $$\Delta \varphi =\frac{\left(2\pi L\Delta n\right)}{\lambda },$$

where L is the laser-gas molecules’ interaction path length and λ is the wavelength of the *probe* light. The unique feature of the PTS is the fact that the *probe* beam wavelength can significantly differ from the *pump* wavelength, hence the PTS signal readout can be achieved using inexpensive and widely available electronic, fiber, and optical components developed for the so-called telecom spectral band (i.e., ~1.55 µm). In addition, when the *pump* light is modulated with a sinewave signal, the spectroscopic information can be conveniently retrieved using the WMS-based technique [47,48,49]. Furthermore, the RI modulation can be also encoded into the frequency deviation of the beat note of the *probe* beam by using the heterodyne detection [49]. In such a configuration, the signal analysis in the frequency, not amplitude domain gives the PTS sensors immunity to the negative influence of, e.g., optical fringes, which results in the baseline-free signal retrieval, hence very high sensor sensitivity. It can be seen from the aforementioned equations that the PTS signal can be linearly enhanced with the increase in the *pump* power density over the interaction path length, however, a perfect overlap between the *pump* and the *probe* beams is mandatory, but not simple to achieve using bulk optics-based components. The perfect solution to this problem comes with the aid of ARHCFs. These fibers are characterized with mode field diameters (MFD) typically in the range of a few tens of micrometers, which means that a small beam size, hence high power density can be efficiently confined and maintained throughout the entire fiber length. Furthermore, the ability to guide light in dissimilar wavelength regions allows ARHCFs to transmit simultaneously both the *pump* and *probe* light in the gas-filled core, providing the perfect overlap between them. So far, several configurations of the PTS sensors utilizing ARHCFs as absorption cells have been developed based on the Mach-Zehnder (MZI) and Fabry–Perot (FPI) interferometer setups and shown to provide an exceptional detection capability [22,26,50,51,52,53,54]. 

### 5.1. MZI PTS in ARHCF

In an MZI-based PTS sensor configuration, the modulation of the RI induced by the gas molecules excitation is observed as the difference in the optical path length (hence the phase difference), that is experienced by the *probe* beam propagating in two arms of the interferometer [55]. In a typical MZI PTS sensor setup, the sensing arm of the interferometer consists of an absorption cell while the second is used as a reference and is free of gas molecules.

**Figure 8:**
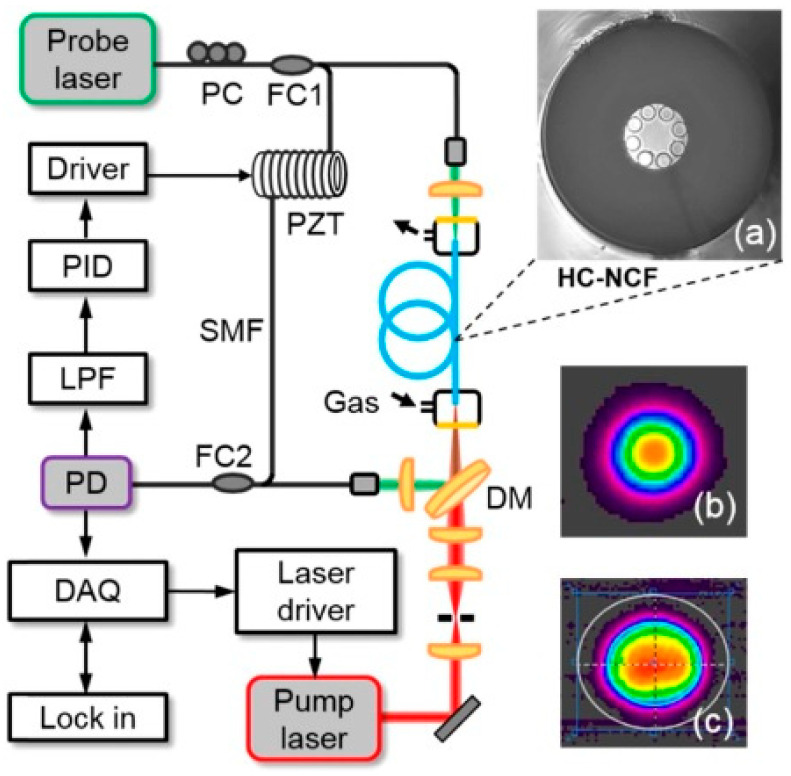
Schematic diagram of the MZI PTS sensor utilizing an HC-NCF as an absorption cell used to detect CO at 2327 nm. The fiber was filled with the gas analyte using gas filling cells placed at both ends. HC-NCF—hollow core negative curvature fiber (ARHCF), PID—proportional-integral-derivative controller, PD—photodetector, LP—Flow-pass filter, FCfiber coupler, PCpolarization controller, PZT—piezo-electric transducer, DM—dichroic mirror. (**a**) Cross-section of the HC-NCF used in the experiment. (**b**) HC-NCF-delivered beam profile. (**c**) Profile of the beam emitted by the *pump* laser. Reprinted with permission from [50] © The Optical Society.

An ARHCF-aided configuration of such a sensor was demonstrated by Yao et al. in [50] and was aimed at detecting CO. The experimental setup of the sensor is presented in Figure 8. In this configuration, the *pump* laser operated at 2327 nm, which corresponds to the R(10) transition of CO in the 2v_1_ band, while the *probe* laser wavelength was set to 1533 nm. The *probe* beam was divided into two arms of the MZI and coupled together with the *pump* light into a 0.85 m long gas-filled hollow-core negative curvature fiber (HC-NCF) placed in the sensing arm. The dichroic mirror in the sensing arm was used to separate the remaining *pump* light from the *probe* beam that contained the information of the induced RI modulation. The MZI was set to operate in the quadrature point by implementing a piezo-electric transducer with a piece of a conventional single-mode fiber coiled on it. The *probe* beams leaving both arms of the MZI are combined using a fiber coupler, and subsequently, the beat note signal was detected by a photodetector. The interferometric signal contained information about the phase change of the *probe* light after passing through the heated gas sample in the fiber core. With the additional sinewave modulation applied to the *pump* laser injection current, the spectroscopic signal was retrieved using the well-known WMS method. The registered signal was free from the intermodal interference noise in the fiber, which allowed the sensor to obtain a normalized noise equivalent absorption coefficient (NNEA) at the level of 4.4 × 10^−8^ cm^−1^ WHz^−1/2^ (90 ppmv), which gives an order of magnitude improvement in comparison to the similar sensor configuration utilizing a hollow-core capillary tube [55].

Further development of ARHCF-aided MZI PTS sensors was reported in [51]. The authors benefited from the unique property of the ARHCFs, which enables them to guide light with low loss both in the near- and mid-IR spectral band. The developed sensor configuration was similar to the one presented in Figure 8, however, the *pump* light was emitted from an ICL operating at 2778.48 cm^−1^, which corresponded to the strong transition of formaldehyde (CH_2_O), while the *probe* beam was delivered from a telecom DFB laser emitting light at 1.56 µm. The gas-laser interaction path in the sensing arm of the MZI was formed by a 1.2 m long ARHCF having an air core with a diameter of 65 µm, which was characterized by the attenuation of 0.6 dB/m and 0.43 dB/m at the *pump* and *probe* wavelengths, respectively. Hence, both wavelengths could be simultaneously coupled into the fiber and transmitted through it with low loss. The ICL was modulated with a sinusoidal signal with a frequency of 8 kHz, which enabled performing WMS-based spectroscopic signal retrieval at the harmonics of the modulation frequency. The authors proved that the demodulation of the photodetector signal at the 1st harmonic (1f detection) provided greater signal amplitude while maintaining a low noise level in comparison to the 2f signal when the sinewave modulation frequency was greater than 6 kHz. Furthermore, the background free behavior of the sensor was maintained utilizing the 1f detection scheme. The obtained SNR for the 1f signal reached 163, which was 2.4 times better compared to the value obtained for the 2f signal. The obtained SNR yielded an MDL of 0.18 ppmv, which corresponds to an NNEA of 4 × 10^−9^ cm^−1^ WHz^−1/2^.

### 5.2. FPI PTS in ARHCF

Another approach to measuring the photothermal effect refers to the application of an FPI, which enables efficient detection of the phase change of the propagating *probe* beam after passing through the heated gas sample, hence experiencing the locally modulated RI. The intensity of the beam exiting the Fabry–Perot cavity can be determined based on the equation [44,56]:

(9) $$I_{t}=I_{0}\frac{1}{1+{\left(\frac{2F}{\pi }\right)}^{2}\sin^{2}\left(\frac{\Delta \varphi }{2}\right)},$$

where I_t_ is the transmitted beam intensity, I_0_ is the intensity of the beam before entering the Fabry–Perot cavity, F is the cavity finesse, Δφ corresponds to the phase change, which can be further defined by [44]:

(10) $$\Delta \varphi =\frac{2\pi }{\lambda_{p}}2\mathrm{nLcos}\theta ,$$

where λ_p_ is the probe laser wavelength, n is the RI, L is the cavity length, and cosθ defines the angle of incidence. It can be seen that due to the photothermal effect inside the cavity, the modulation of the RI has a direct impact on the change in the intensity of the transmitted radiation, which combined with the PTS effect can be used to determine the molecular concertation of the measured sample. Furthermore, in comparison to the homodyne MZI PTS detection scheme, the FPI PTS sensor can achieve long-term repeatability via a non-complex stabilization of the *probe* laser wavelength to the quadrature of the FPI using a proportional-derivative-integral (PID) based approach [52]. Several configurations of the FPI PTS sensors utilizing the ARHCFs have already been demonstrated and will be discussed in this subsection.

**Figure 9:**
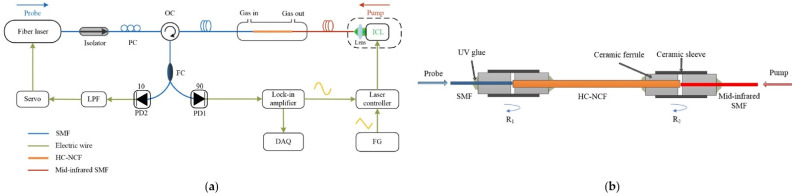
FPI PTS C_2_H_6_ gas sensor based on the use of a mid-IR ARHCF. (**a**) Experimental setup. PC—polarization controller, OC—circulator, ICL—interband cascade laser, FG—function generator, DAQ—data acquisition card, PD—photodetector, LPF—low-pass filter, SMF—single-mode fiber, HC-NCF—hollow core negative curvature fiber. (**b**) The absorption cell is formed by the gas-filled HC-NCF (ARHCF), which is butt-coupled with a conventional SMF and mid-IR SMF. R_1_ and R_2_ indicate the reflections in the FPI cavity formed by the interfaces between the coupling points of these fibers. Reprinted with permission from [26] © The Optical Society.

Chen et al. reported in [26] an FPI PTS gas sensor targeting ethane (C_2_H_6_) at 3.348 µm, which setup is presented in Figure 9a. The absorption cell was formed by a 14 cm long HC-NCF with a core diameter of 65 µm. The molecules of the target gas were excited using an ICL operating at the aforementioned wavelength, while the *probe* beam was delivered from a 1.55 µm fiber laser. The FPI cavity layout is shown in Figure 9b. The *probe* light was coupled into the HC-NCF directly from a conventional single-mode fiber (SMF) using a butt-coupling approach. The same technique was used to couple the *pump* light, however, the ICL beam delivery fiber was an InF_3_ (indium fluoride) mid-IR guiding SMF. The FPI cavity was realized based on the ~4% probe light reflections at the HC-NCF/SMF and HC-NCF/InF_3_ SMF interfaces. The end facets of each fiber were glued into the ceramic ferrules, which mechanically stabilized the coupling between them and were used to deliver the gas sample into the HC-NCF core. The *probe* laser wavelength was locked and stabilized at the quadrature point of the interference fringe using a servo loop, which allowed converting the induced phase change into the intensity change at the output of the FPI. The *pump* wavelength was additionally modulated with a sinewave signal to perform WMS-based signal readout at the 2nd harmonic of the modulation frequency. The remaining *pump* light leaving the HC-NCF was filtered out by the 1.55 µm SMF. The stabilization was mandatory to obtain efficient operation of the sensor and its long-term stability, which was experimentally verified by registering the 2f signal amplitude over an 8 hour period. The system allowed obtaining an MDL of 2.6 ppbv for 410 s integration time, which gives an NEA of 2.0 × 10^−6^ cm^−1^.

**Figure 10:**
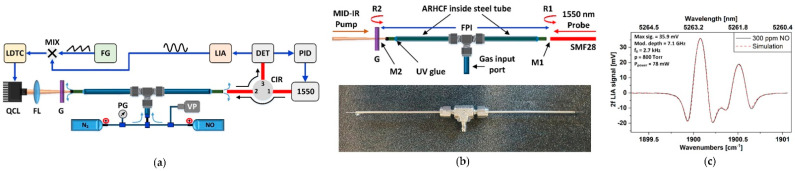
FPI PTS NO sensor utilizing a borosilicate ARHCF. (**a**) Schematic of the sensor setup. LDTC—laser driver, MIX—frequency mixer, FG—function generator, LIA—lock-in amplifier, DET—photodetector, PID—proportional-derivative-integral controller, CIR—circulator, G—germanium window, FL—focusing lens, QCL—quantum cascade laser, VP—vacuum pump, PG—pressure gauge. (**b**) FPI cavity (top). R1 and R2—reflections in the FPI, M1, and M2—FPI mirrors. 25 cm long ARHCF-based absorption cell (bottom) glued into a steel tube inserted into a gas delivery t-junction port. (**c**) Registered 2f signal spectrum from 300 ppmv NO in the ARHCF (black trace) and simulated (red trace). Reprinted from [52] with permission from Elsevier.

Krzempek et al. presented in [52] an FPI PTS sensor configuration aided with a borosilicate ARHCF (shown in Figure 1f), which pushed for the first time the operational wavelength of such sensors beyond the 5 µm range. The experimental setup of the sensor is shown in Figure 10a. In this configuration, the *pump* light was delivered from a QCL operating at 1900.09 cm^−1^, which provided access to a strong transition of NO. The *probe* beam came from a standard telecom DFB laser delivering 1.55 µm output. The *probe* laser wavelength was stabilized using the PID-based approach to keep it operating at the quadrature point of the FPI. The 1550 nm light after passing the circulator was coupled into the ARHCF using the butt coupling technique. After leaving the ARHCF, the *probe* beam was reflected back to the fiber from a germanium window and directed via the circulator to the near-IR photodetector, which combined with a lock-in amplifier allowed 2f WMS-based signal readout. The FPI cavity in the sensor was formed by the *probe* beam reflections from the SMF28 end facet (R1) and the germanium window (R2) as depicted in Figure 10b. The absorption cell was constructed based on a 25 cm long ARHCF with a core size of 122 µm. The ARHCF was filled with the target gas mixture through a set of femtosecond laser processed microchannels, which provided direct access to the core region and eliminated the need of using gas filling cells. The fiber was glued into a steel tube equipped with a gas delivery port, which enabled the authors to efficiently fill the fiber with NO. The registered 2f signal spectrum shown in Figure 10c perfectly matches the simulated signal and confirms the baseline-free characteristic of the sensor. The unique sensor configuration combined with the ARHCF ability to simultaneously guide near- and mid-IR light resulted in an MDL of 11 ppbv for 144 s (NEA ~1.68 × 10^−7^ cm^−1^) integration time with an NNEA of 4.29 × 10^−7^ cm^−1^ WHz^−1/2^. 

FPI PTS sensor aided with ARHCFs was also designed and developed to operate only in the near-IR spectral band, where both the *pump* and the *probe* beam were transmitted within the same low-loss window of the ARHCF. Bao et al. reported in [54] an acetylene (C_2_H_2_) sensor that utilized a 5.5 cm long ARHCF that was *pumped* with 1532.5 nm using a DFB laser. The FPI cavity was similar to the one shown in Figure 9b. The *probe* light at 1551.3 nm was provided by the external cavity diode laser (ECDL) and was separated from the unabsorbed *pump* light in the gas-filled ARHCF using a wavelength division multiplexer (WDM) before being directed onto a photodetector and further analyzed. The spectroscopic signal retrieval was realized using the same approach as described above. The sensor reached an MDL of 2.3 ppbv for 670 s integration time and an NEA of 2.3 × 10^−9^ cm^−1^.

### 5.3. MPD PTS in ARHCF

In the majority, the multimode characteristic of the ARHCFs is not desired in the sensor configurations where the intermodal interference induces a significant increase in the baseline noise in the sensor. However, with a specific sensor design and signal retrieval method, the few-moded behavior of the ARHCF can be very beneficial, especially in combination with the PTS.

**Figure 11:**
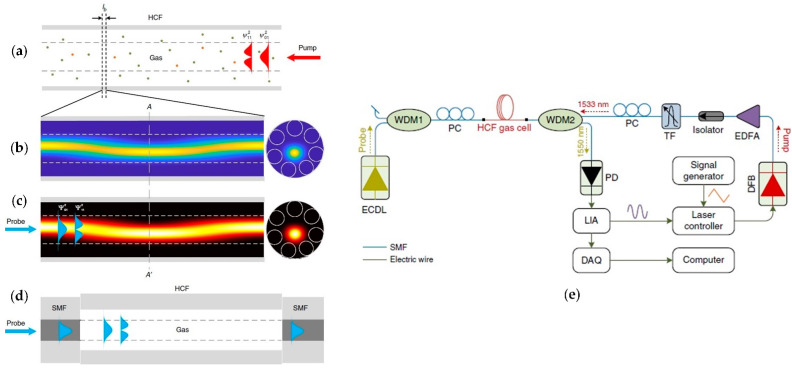
Principle of the MPD-PTS in ARHCF for gas sensing. (**a**) Intensity profiles of the *pump* LP_01_$\left(\psi_{01}^{2}\right)$ and LP_11_$\left(\psi_{11}^{2}\right)$ modes in the gas-filled ARHCF. (**b**) *Pump* intensity change within the modal beat length. (**c**) The temperature change, which induces the RI modulation in the gas-filled ARHCF core while the gas molecules are excited by the *pump* light. It can be seen that the *probe* modes LP_01_$\left(\Psi_{01}^{2}\right)$ and LP_11_$\left(\Psi_{11}^{2}\right)$ are experiencing different RI changes. (**d**) A schematic of the in-line dual-mode interferometer. (**e**) Experimental setup of the MPD-PTS sensor utilizing an ARHCF as a C_2_H_2_ absorption cell. HCF—hollow-core fiber, SMF—single-mode fiber, I_b_—modal beat length, WDM—wavelength division multiplexer, PC—polarization controller, ECDL—external cavity diode laser, TF—tunable optical filter, EDFA—erbium-doped fiber amplifier, LIA—lock-in amplifier, DAQ—data acquisition card Reprinted from [25] with permission from Springer Nature.

Zhao et al. proposed in [25] a novel approach to the PTS technique, the so-called mode-phase-difference photothermal spectroscopy (MPD-PTS), which measures the PT-induced differential phase change of the LP_01_ and LP_11_ modes of the *probe* beam propagating through the gas-filled ARHCF core, which can be expressed by [25]:

(11) $$\delta \varphi =\Delta \varphi_{01}-\Delta \varphi_{11}=k^{*}\left(\eta ,f\right)\left(1-e^{-\alpha \left(\lambda_{\mathrm{pump}}\right)\mathrm{CL}}\right)P_{\mathrm{pump}}\approx k^{*}\left(\eta ,f\right)\alpha \left(\lambda_{\mathrm{pump}}\right){\mathrm{CLP}}_{\mathrm{pump}},$$

where Δφ_01_ and Δφ_11_ are the phase modulation for LP_01_ and LP_11_ modes of the *probe* light, respectively, k* is the differential phase modulation coefficient, which is a function of the fractional *pump* power in the LP_01_ mode—η and sinewave modulation frequency—f of the *pump* light, α(λ_pump_) is the absorption coefficient for the relative concentration of 100%, C corresponds to the target gas concentration, L is the ARHCF length and P_pump_ defines the average *pump* beam power over the fiber length. In principle, the *pump* light transmitted in the gas-filled ARHCF core in the LP_01_ and LP_11_ mode heats the gas sample in a way corresponding to the intensity distribution of each mode as shown in Figure 11a,b. This results in the periodically varying RI modulation following the temperature change trend over the entire core length as presented in Figure 11c. This leads to the coherent mixing of the LP_01_ and LP_11_ *pump* modes with the spatial period corresponding to their beat length [25]. If the *probe* beam is simultaneously coupled to the fundamental mode and the higher-order mode, it experiences the RI modulation induced by the corresponding *pump* light modes. This results in the different phase modulation of each *probe* mode, which can be detected in a sensor configuration based on an in-line dual-mode interferometer as presented in Figure 11d. Here, the hollow-core fiber can act as an absorption cell, which can be filled with the gas analyte through the gaps between its input/output end facets and the SMFs placed at both ends or via a laser-drilled microchannel along its length. The *pump* and *probe* beams were coupled into the ARHCF using the butt coupling approach from SMF, which provided excitation of the LP_01_ and LP_11_ modes. The interferometer cavity was implemented in the setup shown in Figure 10e, which enabled C_2_H_2_ sensing based on the 2f signal readout. The gas molecules were *pumped* at 1532.83 nm using a DFB laser and the induced RI modulation was *probed* with an ECDL operating at 1550 nm. The ARHCF forming absorption cell was designed to operate in the ~1.5 µm wavelength band. The length of the fiber was 4.67 m with an air core diameter of 28 µm. The WDM couplers provided perfect separation of the *probe* beam from the *pump* light before directing it to the photodetector and subsequently to a lock-in amplifier for 2f signal demodulation. The authors have demonstrated that the MPD-PTS sensor can reach an MDL at the level of 15 parts-per-trillion by volume (pptv) for 100 s integration time with an NEA of 1.6 × 10^−11^ cm^−1^. Furthermore, the long-term stability tests of the developed sensor confirmed its excellent robustness, greater in comparison to the ARHCF-aided sensors utilizing the MZI setup.

**Figure 12:**
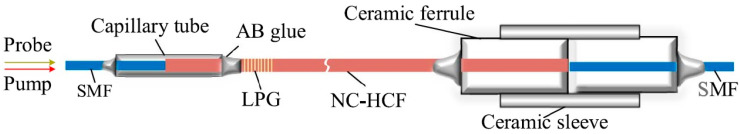
Schematic of the absorption cell formed by an NC-HCF with LPG inscribed in it. The absorption cell forms an in-line dual-mode interferometer. The *probe* and *pump* beams were delivered to and outcoupled from the NC-HCF using the butt-coupling approach with SMFs. Reprinted with permission from [22] © The Optical Society.

The calculations performed by Zhao et al. in [22] have indicated that the differential phase modulation in an MPD-PTS gas sensor reaches its maximum if the *pump* light power is coupled into the LP_01_ mode. However, at the same time, the *probe* light must be transmitted within the LP_01_ and LP_11_ modes simultaneously so the induced RI modulation by the heated inside ARHCF gas molecules can introduce the phase difference between these modes, which can be subsequently analyzed. Excitation of the LP_01_ *pump* mode only and two *probe* modes at the same time is not trivial and almost impossible to realize by offsetting the SMFs with respect to the ARHCF core as it was reported by the Authors in their previous work [25]. According to this, the MPD-PTS gas sensor performance did not reach its maximum. To address this problem the Authors inscribed a long period grating (LPG) in a negative curvature – hollow core fiber (NC-HCF) forming an absorption cell as shown in Figure 12. The LPG enabled the excitation of the LP_01_ and LP_11_ modes at the *probe* wavelength maintaining *pump* light transmission in the LP_01_ mode only. This modification of the sensor resulted in the maximization of the differential phase modulation induced by the photothermal effect and reduced the complexity of the sensor setup. The approach was tested in an experimental configuration of an MPD-PTS detector targeting C_2_H_2_ at 1532.83 nm in an 85 cm long NC-HCF with a core size of ~30 µm. The induced RI modulation was *probed* at 1620 nm. The setup of the sensor and measurement procedure was similar to the described above. The sensor reached an MDL of 600 pptv at 100 s integration time with an NEA of 6.3 × 10^−10^ cm^−1^, maintaining a less complex configuration than reported in [25].

Zhao et al. reported in [53] an MPD-PTS CH_4_ sensor utilizing an ARHCF with an inscribed LPG. The CH_4_ molecules were *pumped* at ~1653.7 nm with the aid of a DFB laser, which was additionally amplified using a fiber-based Raman amplifier to maximize the photothermal effect. The *probe* light at 1550 nm was delivered from a fiber laser equipped with a wavelength stabilization unit. The absorption cell was realized based on a 2.4 m long ARHCF with a core size of ~30 µm, which was characterized by an attenuation of 0.16 dB/m and 0.25 dB/m at the *probe* and *pump* wavelengths, respectively. The sensor setup was built in a configuration similar to the one shown in Figure 11e and the spectroscopic signal retrieval was based on the 2f signal demodulation. In the proposed configuration, a part of the ARHCF forming the absorption cell was placed inside a column oven to investigate the influence of the temperature change on the induced differential phase modulation of the *probe* beam. The performed experiments indicated that the operating point of the dual-mode interferometer (the quadrature point of the interference fringe) is highly sensitive to the temperature deviations, hence a proper compensation of the temperature drift is necessary to improve the sensor stability. It was possible to minimize the photothermal signal variations from ~9.4% to ~2.1% using a linear temperature compensation scheme and obtain an MDL of approximately 4.3 ppbv for 100 s integration time. The NEA coefficient reached the level of 1.6 × 10^−9^ cm^−1^.

### 5.4. PABS in ARHCF

PAS is a spectroscopic technique similar to the PTS, however, in this case, the modulation of the phase of the *probe* light along the gas-molecules interaction path length results from an increase of the local pressure gradient due to gas molecules excitation by the *pump* laser light [57]. When the *pump* laser beam intensity is additionally modulated at a certain frequency, the pressure will change periodically, producing an acoustic wave having a frequency equal to the modulation frequency of the *pump* light [57]. The photoacoustic effect has been widely used in laser-based spectroscopy, delivering an exceptional sensitivity of the PAS-based gas sensors [58,59,60].

**Figure 13:**
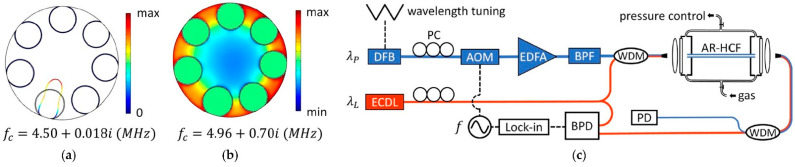
(**a**) A capillary acoustic mode in the ARHCF. (**b**) A core acoustic mode in the ARHCF. f_c_ is the eigenfrequency of each mode. (**c**) Experimental setup of the PABS sensor. DFB—distributed feedback diode laser, ECDL—external cavity diode laser, PC—polarization controller, AOM—acousto-optic modulator, f is the sinewave modulation frequency generator, EDFA—erbium-doped fiber amplifier, BPF—optical bandpass filter, WDM—wavelength division multiplexer, PD—photodetector, BPD—balanced photodetector. Reprinted with permission from [23] © The Optical Society.

The PAS technique was recently combined with gas sensing inside an ARHCF by Zhao et al. in [23]. The authors have shown that it is possible to efficiently excite acoustic modes inside the gas-filled fiber core as a result of the photoacoustic effect and the structure of the ARHCF. This modulates the RI of the gas sample and subsequently the phase of the *probe* beam in the fiber. The interaction between the optical and acoustic modes is explained by means of the Forward Brillouin Scattering (FBS) phenomenon [61]. The silica cladding of the ARHCF forms an acoustic resonator and the gas-filled part of the fiber acts as a region for acoustic wave generation. As a result, the modulated light-excited and heated gas molecules induce an acoustic wave, which is resonantly enhanced inside the ARHCF. When the *probe* light propagates inside the fiber, it experiences a phase modulation, which can be measured using the earlier described dual-mode interferometer method. Since an ARHCF supports capillary and core acoustics modes as shown in Figure 13a,b, the optical LP_01_ and LP_11_ modes differently overlap with them, which introduces different phase modulation of each of these modes. The observed differential phase modulation can be described by the following formula [23]:

(12) $$\Delta \emptyset \left({\Omega \lambda }_{p}\right)=\xi \left(\Omega \right)\alpha \left(\lambda_{p}\right){\mathrm{CL}}_{\mathrm{eff}}P_{p},$$

where Ω is the modulation frequency, λ_p_ is the *pump* wavelength, ξ defines the normalized phase modulation coefficient, α is the absorption coefficient, C is the gas concentration in the ARHCF, L_eff_ is the effective length of the ARHCF (directly depending on the molecular concentration, absorption coefficient and the actual fiber length), and P_p_ is the *pump* power. The gas sensing using PABS in the ARHCF was experimentally verified in the setup depicted in Figure 13c. A DFB laser operating at 1532.83 nm was used as the *pump* source for C_2_H_2_ molecules excitation in a 30 cm long ARHCF-based absorption cell. The *pump* light was modulated with a sinewave signal at the frequency Ω, which corresponds to the frequency of the selected acoustic mode with the aid of an acousto-optic modulator (AOM). The *probe* beam was delivered from an ECDL and simultaneously coupled with the *pump* beam into the gas-filled ARHCF. Via the lateral offset coupling to the ARHCF, it was possible to excite LP_01_ and LP_11_ modes, hence forming a dual-mode interferometer. The ARHCF was filled with the measured gas via a gas filling cell. The optical signal was directed to a balanced photodetector to minimize the intensity noise and subsequently demodulated at f = Ω/2π using a lock-in amplifier. The system was characterized to provide an MDL of 8 ppbv for 100 s integration time. Operation of the system at frequencies in the range of MHz allows it to minimize the negative influence of the 1/f noise, which is not obtainable in PTS-based gas sensors.

## 6. Summary

In this review, recent advancements in the ARHCF-based gas sensors were presented and discussed. It was shown that the properly designed ARHCFs could be successfully used as low volume, compact, and long absorption cells in sensing systems utilizing various laser spectroscopic techniques, i.e., TDLAS, WMS, PTS (in several dissimilar configurations), and PABS. The ARHCF absorption cells forming the gas molecules’ interaction path length within the sensor can be efficiently and at a reasonably fast time filled with the target gas mixture using an overpressure induced gas flow, which results in an acceptable response time of such a sensor. A summary of the performance of the selected ARHCF-aided gas sensors is presented in Table 2.

The ARHCF-aided gas sensors utilizing the TDLAS technique indeed proved their capability to detect molecules of various gases in a simple and non-complex way, however, did not provide sufficient suppression of the background noise resulting from, i.e., intermodal interference. This drawback severely limits the sensitivity and long-term stability of such sensors, hence different spectroscopic techniques have to be combined with ARHCFs to fully benefit from the connection of both.

The combination of ARHCFs with the WMS technique unquestionably provides a significant improvement in the noise level reduction in the ARHCF-assisted gas sensor configurations, which directly leads to a better sensor sensitivity. However, such sensors are still not immune to the uncontrolled fluctuations of the registered signal. Since especially short lengths of the ARHCFs are characterized by the presence of higher-order modes or longer pieces from the non-uniformity of the structure, the amplitude-based signal retrieval is sensitive to the parasitic changes of the analyzed light intensity, which significantly impacts the overall performance of this type of gas sensors. Hence, not only proper optimization of the fiber structure but also a change of the gas sensing method can provide the desired improvement in the operation of the ARHCF-based sensors.

The other approach for gas sensing aided from ARHCFs comes with the PTS technique, where the spectroscopic signal retrieval is based on the analysis of the *probe* light phase modulation induced by a local change in the RI due to heating of gas molecules. The phase modulation can be precisely investigated based on the interferometric signal measurement. The ARHCFs were implemented in the PTS sensors utilizing MZI, FPI, and MPD sensor setups. The main drawback of the MZI PTS gas sensors is the necessity of actively and precisely stabilizing the sensor’s setup to maintain its operation at the quadrature point of the interferometer. This is not simple to realize, and the long-term stability of such sensors is very difficult to be obtained, which severely limits their ability to operate in real application conditions. The less complex sensor design is delivered by the application of an FPI. In an FPI PTS gas sensor utilizing ARHCFs, stabilizing the cavity at quadrature can be realized in a much more accessible and reliable way, by stabilizing the wavelength of the *probe* beam, e.g., with an aid of PID-based technique. With this technique, the sensitivity of the ARHCF-assisted PTS sensors is improved in comparison to the sensors based on the MZI. Until now, the most sensitive configuration of the ARHCF-based gas sensors was based on the MPD PTS technique. The MPD PTS sensors were shown to provide significantly better long-term stability and immunity to the negative influence of external factors than, e.g., MZI PTS. However, it can be assumed that even simple WMS-based sensors aided with several tens of meters long ARHCFs could provide comparable or even better sensitivity benefiting from long interaction path lengths. The main requirements which have to be fulfilled are the pure single-mode guidance of the ARHCF and the uniformity of its structure, which both are mandatory to eliminate the fringe noise and the parasitic fluctuations of the signal amplitude. Furthermore, the application of heterodyne signal retrieval in the MZI PTS configurations instead of the homodyne technique will result in the possibility of analyzing the signal in the frequency, not amplitude domain which could provide the ultimate immunity of the sensor to the fringe noise (resulting from the intermodal interference in the ARHCFs) and the amplitude noise (e.g., residual amplitude modulation) [49].

A very interesting approach to gas sensing inside the ARHCFs was presented in [23], where the sensing system was based on the PABS technique. The interaction between the fiber-supported acoustics and optical modes enables signal retrieval in the MHz frequency range, which minimizes the 1/f noise. It was stated that with the further optimization of the fiber structure, hence the structure of the formed in it an acoustic resonator, ARHCF-based PABS sensor could deliver significantly greater sensitivity than reported by the authors.

The ARHCF-based gas sensors have already proved their excellent suitability for this application and opened a way to the new branch of sensitive, low-volume, and versatile detectors. It is expected that further development of the hollow-core fiber technology will result in the possibility of fabricating several tens of meters long fibers, maintaining uniform structure along their entire length, which will enable low-loss and single-mode transmission not only in the near- but also in the mid-IR spectral band. The utilization of different than fused silica materials, such as telluride or chalcogenide glasses should extend the operational wavelength range of these fibers to the spectral bands above 5.26 µm, hence allowing the detecting of various gases over a significantly broader than currently available range. The improvement in the transmission properties of the ARHCFs should also allow them to be successfully used in the broadband spectroscopy applications, i.e., in the frequency comb spectroscopy [62], which will enable an in-depth analysis of the complex mixture of gaseous substances, maintaining a low-volume of the sensing unit with high detection capability. Currently, the ARHCF-based gas sensors are in majority realized in laboratory conditions, which results in their still large size (despite the low-volume formed by the absorption cells) in comparison to field-deployable bulk-optics based sensors [63] and most commonly used in real-life applications non-optical gas detectors [64,65]. However, further minimization of the electronic components, accompanied with optics-free light coupling into the fiber and the possibility of bending the fiber with bend radius in the range of a few centimeters [29,66] should result in a significant reduction in the size of ARHCF-aided gas sensors, even beating the dimensions of the sensors utilizing multipass cells delivering several tens of meters long optical path lengths. Moreover, proper laser-based modification of the fiber structure, enabling loss-free access to the fiber core for gas filling purposes [67], should result in pure diffusion-based gas exchange in such sensors, significantly enhancing their versatility and usefulness together with further reduction in the overall size of the entire sensing unit. The combination of a low-volume, non-complex design, selectivity, excellent detection capability, and calibration-free operation should lead the ARHCF-aided gas sensing approach to the development of the sensors that could in future form a new branch of gas spectrometers with the parameters comparable or even beating the currently used devices.

## Figures and Tables

**Table 1:** Comparison of the performance of the HCFs used in gas sensing applications.

Fiber Type	Wavelength	Light Guidance	Core Size Loss @ 3.4 µm	Loss @ ~3 µm	Min. Gas Loss Filling Time for ~1 m Fiber
ARHCF	up to 5.26 µm [20]	Single-mode with proper fiber structure [20]	84 µm [8]	0.03 dB/m [8]	5 s [21]
Kagome HCF	up to 3.4 µm [18]	few-moded [18]	116 µm [18]	~0.1 dB/m [18]	<10 s [18]
HC-PBGF	up to 3.4 µm [9]	few-moded [9]	40 µm [9]	2.6 dB/m [9]	1200 s [16]

**Table 2:** Comparison of the performance of the ARHCF-based gas sensors.

Configuration	Gas	Wavelength	ARHCF Length	Filling Time	MDL	NEA [cm^−1^]	NNEA [cm^−1^ WHz^−1/2^]	Integration Time
TDLAS [21]	CO_2_	2004 nm	1.35 m	5 s	1.5%	-	-	-
TDLAS [38]	CO	2326.8 nm	0.85 m	5 s	13 ppmv	5.2 × 10^−6^	-	-
TDLAS [35]	N_2_O	3599.2 nm	1.20 m	-	-	2.5 × 10^−7^	-	40 s
TDLAS [24]	NO	5262.9 nm	0.21 m	0.3 s	1.2 ppmv	2.1 × 10^−5^	-	-
WMS [21]	CO_2_	2004 nm	1.35 m	5 s	5 ppmv	7.4 × 10^−7^	-	3 s
WMS [38]	CO	2326.8 nm	0.85 m	5 s	0.4 ppmv	1.6 × 10^−7^	-	30 s
WMS [24]	NO	5262.9 nm	0.21 m	0.3 s	6 ppbv	1.0 × 10^−7^	-	30 s
WMS [27]	N_2_O	4537.8 nm	3.20 m	23 s	5.4 ppbv	3.7 × 10^−7^	-	1 s
WMS [20]	NO	5262.9 nm	1.15 m	9 s	20 ppbv	2.0 × 10^−5^	-	70 s
WMS [8]	CO_2_	1574 nm	1.0 m	-	144 ppmv	1.17 × 10^−7^	-	1.5 s
WMS [8]	CH_4_	3334 nm	1.0 m	19 s	24 ppbv	1.6 × 10^−7^	-	40 s
MZI PTS [50]	CO	2327 nm	0.85 m	-	90 ppmv	-	4.4 × 10^−8^	-
MZI PTS [51]	CH_2_O	3599.1 nm	1.20 m	-	0.18 ppmv	-	4.0 × 10^−9^	-
FPI PTS [26]	C_2_H_6_	3348.15 nm	0.14 m	300 s	2.6 ppbv	2.0 × 10^−6^	-	410 s
FPI PTS [52]	NO	5262.9 nm	0.25 m	63 s	11 ppbv	1.68 × 10^−7^	4.29 × 10^−7^	144 s
FPI PTS [54]	C_2_H_2_	1532.5 nm	0.055 m	52 s	2.3 ppbv	2.3 × 10^−9^	-	670 s
MPD PTS [25]	C_2_H_2_	1532.83 nm	4.67 m	-	15 pptv	1.6 × 10^−11^	-	100 s
MPD PTS [22]	C_2_H_2_	1532.83 nm	0.85 m	-	600 pptv	6.3 × 10^−10^	-	100 s
MPD PTS [53]	CH_4_	1653.7 nm	2.40 m	-	4.3 ppbv	1.6 × 10^−9^	-	100 s
PABS [23]	C_2_H_2_	1532.83 nm	0.30 m	20 s	8 ppbv	-	-	100 s

## Data Availability

Not applicable.

## Nomeclature

**List of Acronyms**	**Definition**
AOM	acousto-optic modulator
ARHCF	Antiresonant Hollow-Core Fiber
ARROW	Antiresonant Reflecting Optical Waveguiding
CLaDS	Chirped Laser Dispersion Spectroscopy
CW	continuous wave
DFB	distributed feedback diode laser
DFG	difference frequency generation
ECDL	external cavity diode laser
FBS	Forward Brillouin Scattering
FPI	Fabry-Perot interferometer
FWHM	full width at half maximum
HCF	hollow-core fiber
HC-NCF	hollow-core negative curvature fiber
HC-PBGF	hollow-core photonic bandgap fiber
ICL	interband cascade laser
LPG	long period grating
MDL	minimum detection limit
MFA	minimum fractional absorption
MFD	mode field diameter
mid-IR	mid-infrared
MPD-PTS	Mode-phase-difference photothermal spectroscopy
MZI	Mach-Zehnder interferometer
NC-HCF	negative curvature–hollow core fiber
NEA	noise equivalent absorption
near-IR	near-infrared
NNEA	normalized noise equivalent absorption
PABS	Photoacoustic Brillouin Spectroscopy
PAS	Photoacoustic Spectroscopy
PID	proportional-derivative-integral
ppbv	parts-per-billion by volume
ppmv	parts-per-million by volume
PTS	Photothermal Spectroscopy
pptv	parts-per-trillion by volume
QCL	quantum cascade laser
RI	refractive index
SMF	single-mode fiber
SNR	signal-to-noise ratio
TDLAS	Tunable Diode Laser Absorption Spectroscopy
WDM	wavelength division multiplexer
WMS	Wavelength Modulation Spectroscopy
